# Chimeric Antigen Receptors for T-Cell Malignancies

**DOI:** 10.3389/fonc.2019.00126

**Published:** 2019-03-05

**Authors:** Lauren D. Scherer, Malcolm K. Brenner, Maksim Mamonkin

**Affiliations:** ^1^Texas Children's Hospital, Houston, TX, United States; ^2^Center for Cell and Gene Therapy, Baylor College of Medicine, Houston, TX, United States; ^3^Houston Methodist Hospital, Houston, TX, United States; ^4^Department of Pathology and Immunology, Baylor College of Medicine, Houston, TX, United States

**Keywords:** T-cells, chimeric antigen receptors (CARs), T cell malignancy, non-Hodgkin lymphoma, immunotherapy

## Abstract

Development of chimeric antigen receptor (CAR)-modified T cells for the treatment of T-lineage leukemia and lymphoma has encountered several unique challenges. The most widely expressed tumor antigen targets for malignant T cells are often also expressed on non-malignant T cells. Transducing T cells with CARs targeted to these shared antigens can therefore promote over-activation or fratricide of CAR T cells, reducing their therapeutic potency. If fratricide is resolved, clinical CAR T cell activity may eliminate normal T-cell subsets and cause temporary immunosuppression. In this review, we summarize the preclinical development of CAR-based therapies for T-cell malignancies and discuss strategies to minimize toxicities associated with on-target fratricide and off-tumor activity.

## Introduction

T-cell malignancies are a heterogeneous group of disorders of clonal growth and T cell dysfunction broadly grouped into T-cell lymphomas (TCLs) and T-cell leukemias, with mature and precursor subtypes. Initial treatment with conventional chemotherapy often results in profound toxicity with insufficient efficacy to sustain remission (1). While intensifying chemotherapy has improved initial response rates in adult and pediatric T cell leukemia up to 90% (2–4), as many as two-thirds of these patients relapse, and salvage chemotherapy has limited success (5, 6). In patients with relapsed or chemotherapy refractory T-cell malignancies, outcomes are poor, and there are limited effective, tolerable therapies. For the 10–50% of patients achieving complete remission (CR) after salvage chemotherapy, the only curative option remains allogeneic stem cell transplantation (ASCT). However, cure rates for ASCT remain at 30% or less and not all CR patients are eligible for transplant (7). Other T-cell malignancies, including cutaneous and peripheral T-cell lymphoma (CTCL and PTCL, respectively), have even lower initial response rates to chemotherapy, and even in the responders, progression-free survival remains at 40–50% (8). Thus, despite progress in treating T-cell malignancies, there remains a need for new, targeted regimens to improve outcomes, particularly for relapsed and refractory patients.

Immunotherapy has produced unprecedented responses in patients with otherwise refractory and relapsed malignancies. Many of these therapies, however, have had only modest benefit in T-cell malignancies and are therefore reserved for selected cases. For example, checkpoint inhibitors had excellent success in a subset of T-cell lymphomas (ENKL), but produced only marginal benefit in other TCLs with an overall response rate of 30–40% (9). Further, these agents actually increased disease progression due to tumor activation in adult T-cell lymphocytic leukemia (ATLL) (10). Antibody therapy has had similarly limited benefit in subsets of TCL. For instance, mogamulizumab, a humanized anti-CCR4 monoclonal antibody, demonstrated limited responses in PTCL-NOS, and some benefit in CTCL (11). However, overall response rates of T cell malignancies to immunotherapy remain modest.

Chimeric antigen receptor (CAR) T cells are among the most promising immunotherapies for cancer, producing remarkable response rates in patients with B-lymphoid malignancies. Consequently, T cells transduced with CARs targeting CD19, an antigen widely expressed in B-cell leukemia and lymphoma, became the first licensed T cell therapy for cancer (12–14). The success of CD19 CAR T cells in treating relapsed and refractory B-cell malignancies has led to broader application of CARs to other tumors. Given similarities between in B and T lymphoid malignancies, extending CAR T cell therapy to these diseases seems straightforward. However, CAR T cell therapies for T cell malignancies have proven difficult to develop and implement. Shared expression of many target antigens on the engineered T cells can result in CAR T cell fratricide during manufacture and the ablation of normal peripheral T-cell subsets after administration, resulting in profound immune deficiency.

Several groups have developed engineered cell-based approaches to address the above challenges, including targeting more restricted T cell antigens like CD30, CCR4, and CD37 (Table 1). This review will cover efforts to target these antigens, the potential value and limitations of these approaches, and the future directions of immunotherapy for T cell malignancies.

**Table 1:** ^♢^Targetable antigens in T-cell malignancies and their expression in normal and malignant cells.

**Antigen**	**Frequency in T-cell malignancies**		**Expression in normal tissues**	**Clinical application**	**References**
	**T-ALL/Ly**	**TCL**			
**PAN-T CELL ANTIGENS**					
CD5	90%	85% (PTCL-NOS)	T cells, thymocytes, B-1 cells	Clinical Trial	(15–17)
		96% (AITL)		NCT03081910°	
		26–32% (ALCL)			
		36% (NK-T)			
		85% (ATLL)			
		91% (CTCL)			
CD7	>95%	50% (PTCL-NOS)	T cells, thymocytes	Clinical Trial	(15, 17–21)
		57% (AITL)	NK cells	NCT03690011^∇^	
		32–54% (ALCL)		NCT02742727^±^	
		79% (NK-T)			
		25% (ATLL)			
		18% (CTCL)			
CD3	33%	60–66% (PTCL-NOS)	Mature T cells		(22)
		71% (AITL)			
		32–40% (ALCL)			
		36%^*^ (NK-T)			
		80% (ATLL)			
		91% (CTCL)			
**ANTIGENS WITH RESTRICTED EXPRESSION**					
CD30	17%^*^	16% (PTCL-NOS)	Activated T and B cells	Clinical Trial	(22–24)
		32–50%^*^ (AITL)		NCT02917083°	
		93% (ALCL)		NCT02690545°	
		64%^*^ (NK-T)		NCT03602157°	
		39% (ATLL)		NCT03383965°	
		18% (CTCL)		NCT03049449°	
				NCT02958410°	
TRBC1	7–11%	27% (PTCL-NOS)	~35% of T cells	NCT03590574°	(20, 25–28)
(TCR)		34% (AITL)			
		25% (ALCL)			
CCR4	~0%	34% (PTCL)	Tregs, Th2 and Th17 cells		(29–32)
		88% (ATLL)	Platelets		
		31–100% (CTCL)	Kidney		
CD4	12%	60% (PTCL-NOS)	CD4^+^ T cells		(33, 34)
		86% (AITL)	Some monocytes and		
		63% (ALCL)	Dendritic cells		
		29%^*^ (NK-T)			
		94% (ATLL)			
		92% (CTCL)			
CD37	~0%	82%	Mature B cells		(35, 36)
			At a low level in plasma cells		
			Low levels in dendritic cells		

* *Only partial positivity observed in malignant cells. ALCL, anaplastic large cell lymphoma; PTCL-NOS, peripheral T cell lymphoma-not otherwise specified; AITL, angioimmunoblastic T-cell lymphoma; CTCL, cutaneous T-cell lymphoma; ATLL, adult T-cell lymphoma and leukemia; T-ALL/LBL, T-cell acute lymphoblastic leukemia or lymphoma*.

° *Clinical trial status: recruiting*.

∇ *Clinical trial status: not yet recruiting patients*.

± *Clinical trial status: unknown*.

♢ *Gorczyca (37) and Karube et al. (38)*.

### Targeting Pan-T Cell Antigens

#### CD5

The first published CAR specifically designed to treat T cell malignancies targeted CD5 (39), one of the characteristic surface markers of malignant T cells. CD5 expression is detected in approximately 80% of T-cell acute lymphoblastic leukemia (T-ALL) and lymphoma (15, 16), as well as in some B-cell lymphomas. CD5 expression by normal cells is restricted to thymocytes, peripheral T cells and a minor subpopulation of B-lymphocytes (40, 41) and it is not expressed by other hematopoietic cells. CD5 functions as a negative regulator of T-cell receptor signaling (42–44) and is implicated in promoting the survival of normal and malignant human lymphocytes (45–48). CD5 is rapidly internalized upon binding with an antibody; this property of the CD5 molecule was used to deliver an immunotoxin into malignant T cells using a toxin-conjugated CD5 antibody. Administration of the modified CD5 antibodies depleted malignant T cells in patients with cutaneous T-cell lymphoma and T-ALL (49–51). These clinical trials demonstrated low-level toxicities due to non-specific uptake of the toxin conjugate in liver and kidneys, but no severe, irreversible adverse events attributable to “on-target, off-tumor” activity, demonstrating the tolerability of targeting such a widely expressed T cell marker and suggested that CD5 CART could be used to safely target T-cell malignancies.

Expression of a second generation CD5 CAR with the CD28 costimulatory domain resulted in the loss of detectable CD5 expression on the surface of T cells (39). In these studies, rapid disappearance of surface CD5 molecules was also observed in normal T cells *in trans* upon coculture with T cells expressing a truncated non-cytotoxic CD5 CAR. This loss could be attributed to rapid capping and internalization of CD5 molecules upon binding the CAR, or to blocking the CD5 antibody epitope. The loss of the target antigen quickly rendered CD5 CAR T cells resistant to fratricide and enabled them to expand *ex vivo*. CD5 CAR T cells had robust cytotoxicity against CD5^+^ T-cell lines and primary tumor cells *in vitro* and protected mice from systemic leukemia progression in two xenograft models of human T-ALL. Despite the high activity against malignant cells, CD5 CAR T cells had limited toxicity against normal activated CD5^+^ T cells, likely due to the higher inherent resistance of normal T cells to their own cytotoxic mechanisms. These promising results suggested that the activity of CD5 CAR T cells *in vivo* would selectively affect malignant cells, with limited damage to the non-malignant T-cell compartment. CD5 CAR T cells are currently being evaluated in patients with refractory or relapsed T-ALL and T-cell lymphoma at Baylor College of Medicine (MAGENTA study, NCT03081910).

#### CD7

CD7 is a transmembrane glycoprotein normally expressed by the majority of peripheral T-cells and NK cells and their precursors, serving as a co-stimulatory protein aiding T-cell activation and interaction with other immune subsets (18, 52). More than 95% of lymphoblastic leukemias and lymphomas, as well as some peripheral T-cell lymphomas, express CD7 (15, 37). In murine models, T cells lacking CD7 demonstrated largely unperturbed development, homeostasis, and protective function (53, 54). As CD7 does not appear to make a pivotal contribution to the function of peripheral T cells, it is a promising target for CAR T cell therapy. Like CD5, CD7 was previously evaluated as a target for monoclonal antibody (mAb) as an immunotoxin-loaded antibody for patients with T-cell malignancies. The mAb conjugate produced no severe CD7-directed toxicities, but tumor responses were modest, likely due to limited activity of murine antibodies in human patients (55).

Three groups, including our own, have recently reported the development and activity of CD7-specific CARs in preclinical models of T-cell malignancies (56–58). In all of these studies, the expression of a CD7 CAR on T cells resulted in fulminant fratricide precluding the *ex vivo* expansion of CAR-modified T cells. To minimize fratricide and enable the expansion of CD7 CAR T cells, surface expression of CD7 must be disrupted, either by editing the CD7 gene (56, 57) or by blocking CD7 protein trafficking to the cell surface (58). Abrogating CD7 expression by either mechanism did not affect proliferation or short-term effector function of T cells and preserved their anti-tumor activity (56). After removing CD7 from the cell surface, CD7 CAR T cells expanded and exerted potent anti-leukemic activity *in vitro* and *in vivo* against primary CD7^+^ T-ALL and lymphoma. We also observed toxicity of CD7 CAR T cells against peripheral CD7^+^ T and NK cells, indicating these subsets will also be targeted in patients. A Phase 1 clinical trial evaluating CD7 CAR expressed on autologous CD7-edited T cells in patients with CD7^+^ T-cell malignancies is in preparation at Baylor College of Medicine (CRIMSON study NCT03690011).

#### CD3

The majority of mature T-cell lymphomas and a small subset of T-cell acute lymphoblastic leukemias (T-ALL) express components of the TCR, such as CD3 and TCRa/b chains, on the cell surface. CD3 is expressed only in the hematopoietic system and its expression is limited to T cells and thymocytes (22). CD3ε-specific immunotoxin-loaded monoclonal antibodies have been evaluated in patients with T-cell lymphoma and were well-tolerated but short-lived, producing partial remissions in some patients (59). Like the CD7-directed approach, expression of a CD3-specific CAR on T cells led to self-targeting and required removal of surface CD3 expression. After mitigating fratricide, CD3 CAR T cells expanded and cleared CD3^+^ tumors *in vitro* and in mouse xenografts (60, 61).

This and other TCR-targeting approaches (62, 63) have to be advanced with caution, however, as the expression of a TCR/CD3-specific CAR on the cell surface of infused T cells may promote TCR cross-linking on normal T cells, in a similar way to bispecific T-cell engagers containing a CD3-binding domain. This potential cross-linking could lead to T cell activation and immediate rejection of the infused product, a possibility that will need evaluation in clinical studies.

### Targeting More Restricted T-Cell Antigens

#### CD30

One of the first CAR T cells with the potential to target T cell malignancies was designed as therapy for Hodgkin's lymphoma and other CD30^+^ malignancies, including anaplastic large cell lymphoma (ALCL). CD30 expression is induced on T cells after antigenic stimulation and can be found in some T cell malignancies, including T-ALL and anaplastic large cell lymphoma (ALCL) (24). Brentuximab vedotin is a CD30-targeted immunoconjugate that was effective in a phase II trial of relapsed, refractory peripheral T cell lymphoma and AITL (64), both of which exhibit high expression of CD30. Brentuximab also produced responses in CD30^+^ CTCL, but of only limited duration (65).

When the antigen-binding domain of a CD30 mAb is expressed as part of a CAR with a CD28 costimulatory domain on T cells there is improved cytotoxicity in both preclinical and early phase clinical studies compared to monoclonal antibody therapy, producing complete clinical responses even in patients who had received prior brentuximab therapy (66–69). Ramos et al. demonstrated the safety and efficacy of CD30 CAR T cells in their Phase I trial of nine patients with relapsed, refractory, heavily pre-treated Hodgkin lymphoma (HL) and EBV negative, CD30^+^ ALCL. In that study, one of six patients with relapsed active HL entered complete remission that lasted over 3 years and three additional patients had transient stable disease. No significant toxicities were observed (66). In a separate phase I clinical trial of T cells modified with a CD30 CAR incorporating a 4-1BB costimulatory domain (67), 18 patients (17 with HL, 1 with ALCL) were treated with CD30 CAR T cells following cytoreductive chemotherapy. Seven patients had a partial response and six had stable disease in response to therapy. Adverse events (AEs) were more notable in this study, but were limited primarily to clinical toxicities, including rash and transient liver inflammation, rather than increased susceptibility to infection. Given that pre-infusion cytoreduction has been reported to potentially increase CAR T cell efficacy, Ramos et al. added lymphodepletion prior to CAR T cell infusion in their subsequent trial (68, 69). Results from this study demonstrated a substantial increase in CD30 CAR T cell expansion and efficacy, with limited AEs. Of the nine patients initially infused, six had a complete response lasting >6 weeks to >6 months. Similarly, Grover et al. infused nine patients with CD30^+^ malignancies after cytoreduction and six patients had a CR that was maintained over 1 year in four. There were no serious AEs reported.

The above clinical trials were the first to demonstrate that CAR T cell therapy can safely target T cell antigens without perturbing systemic T cell immunity. Though a minority of T cell tumors express CD30, these therapies may still play a role in combinatorial therapy for T cell malignancies.

#### TRBC1

While most T-ALL do not express surface TCR, many lymphoma cells are TCR-positive (70) and some PTCL may depend on TCR-associated signaling for lymphomagenesis and survival (71). Thus, the TCR is an attractive target in peripheral T-cell lymphoma. Since targeting the TCR as a pan-T cell marker may eliminate normal T cells, Maciocia et al. took advantage of the clonal nature of malignant T cells and the fact that T cells exclusively express only one of the two genes encoding a TCR beta chain constant region: either TRBC1 or TRBC2. Because about half of TCR^+^ T-cell lymphomas solely express TRBC1, and normal T cells express only one or the other, using a TRBC1-directed CAR could specifically eliminate malignant cells in patients with TCR^+^ disease while sparing normal T cells expressing TRBC2 (27, 28). A similar approach has been evaluated in B-cell malignancies using CARs targeting the kappa light chain of the BCR, leading to the elimination of clonal kappa^+^ malignant B cells and sparing normal lambda light chain-expressing B cells (72). Investigators have shown that TRBC1 CAR T cells specifically eliminate malignant T cell lines expressing TRBC1 while sparing TRBC2-expressing T cells (62). Therefore, this and the reciprocal TRBC2-specific approaches may benefit patients with peripheral T-cell lymphoma as well as a small fraction of T-ALL patients, without inducing complete T-cell ablation. Like CD3-directed CARs, however, the potential crosslinking of normal TCR on T cells by TRBC1 CAR T cells may decrease persistence of the infused T cells and limit their anti-tumor activity. This approach is being evaluated in a Phase I clinical study at University College London (AUTO4 protocol, NCT03590574).

#### CCR4

The chemokine receptor CCR4 may also be a valuable target in patients with T-cell malignancies, as its normal expression in healthy individuals is primarily in regulatory (Tregs), Th2, and Th17 T cells, as well as platelets (29, 73–77). However, CCR4 is over-expressed by malignant cells in many hard-to-treat tumors and may contribute to tumorigenesis and progression. Expression is almost ubiquitous in adult T-cell leukemia (ATLL) (30, 31), as well as in the majority of cutaneous T cell lymphomas (PTCLs, CTCL, mycosis fungoides/Sezary Syndrome) (78, 79), and ALCL (80–82). The FDA-approved CCR4 monoclonal antibody, mogamulizumab, had modest activity in clinical trials against ATLL and CTCL (11, 83), and CCR4 CAR T cells showed *in vitro* and *in vivo* activity against some but not all malignant T cell lines expressing high levels of CCR4 in preclinical studies (84). The consequences of targeting CCR4-expressing Tregs and platelets require careful evaluation, however, as aplasia of these normal cell populations may lead to significant toxicities.

#### CD4

CD4 is expressed by the majority of mature T-cell lymphomas, as well as by a subset of T-ALLs, but only on a subset of mature T cells and some myeloid cells, making it another potential target for immunotherapy. CD4 monoclonal antibodies have been used clinically for over 25 years, with applications in multiple autoimmune disorders, as well as cutaneous and peripheral T cell lymphomas (85–88). These mAb studies have demonstrated that CD4 cell depletion is well-tolerated and reversible, often without clinical evidence of immunosuppression. Given the safety of CD4 mAbs, Pinz et al. expressed an anti-CD4 CAR in activated cells, which resulted in fratricide of CD4^+^ T cells and enrichment for CD8^+^ CD4 CAR T cells. While these studies showed that CD8^+^ CD4 CAR T cells were cytotoxic against CD4^+^ tumor cell lines (34), the most effective ratio of CD4 to CD8 T cells for adoptive transfer remains the subject of ongoing investigation.

#### CD37

CD37 is a transmembrane protein expressed primarily by mature B cells, with lower expression in plasma cells and dendritic cells (89, 90). The molecule is also highly expressed in some subsets of T cell lymphoma, including AILTs, ALK^+^, or ^−^ALCL, adult T-cell leukemias, PTCL NOS, and all extranodal NK/T-cell lymphomas, nasal type. CD37 has a role in T-cell proliferation, neutrophil adhesion, and migration of dendritic cells into lymph nodes (91–94). A phase I dose escalation trial of the CD37 monoclonal antibody drug conjugate (AGS67E) showed good tolerance and partial responses in some patients with cutaneous T cell lymphoma and peripheral T cell lymphoma (95). Consistent with these data, T cells expressing a CD37-specific CAR demonstrated limited fratricide and potent anti-tumor activity in preclinical models of CD37^+^ T-cell lymphoma (96). Although CD37 is present on only a minority of T cell tumors, this same limited expression on normal T cells will decrease the risk of T-cell aplasia.

### Strategies to Limit Fratricide and Exhaustion of T-Cells Expressing T-Lineage Specific CARs

**Figure 1:**
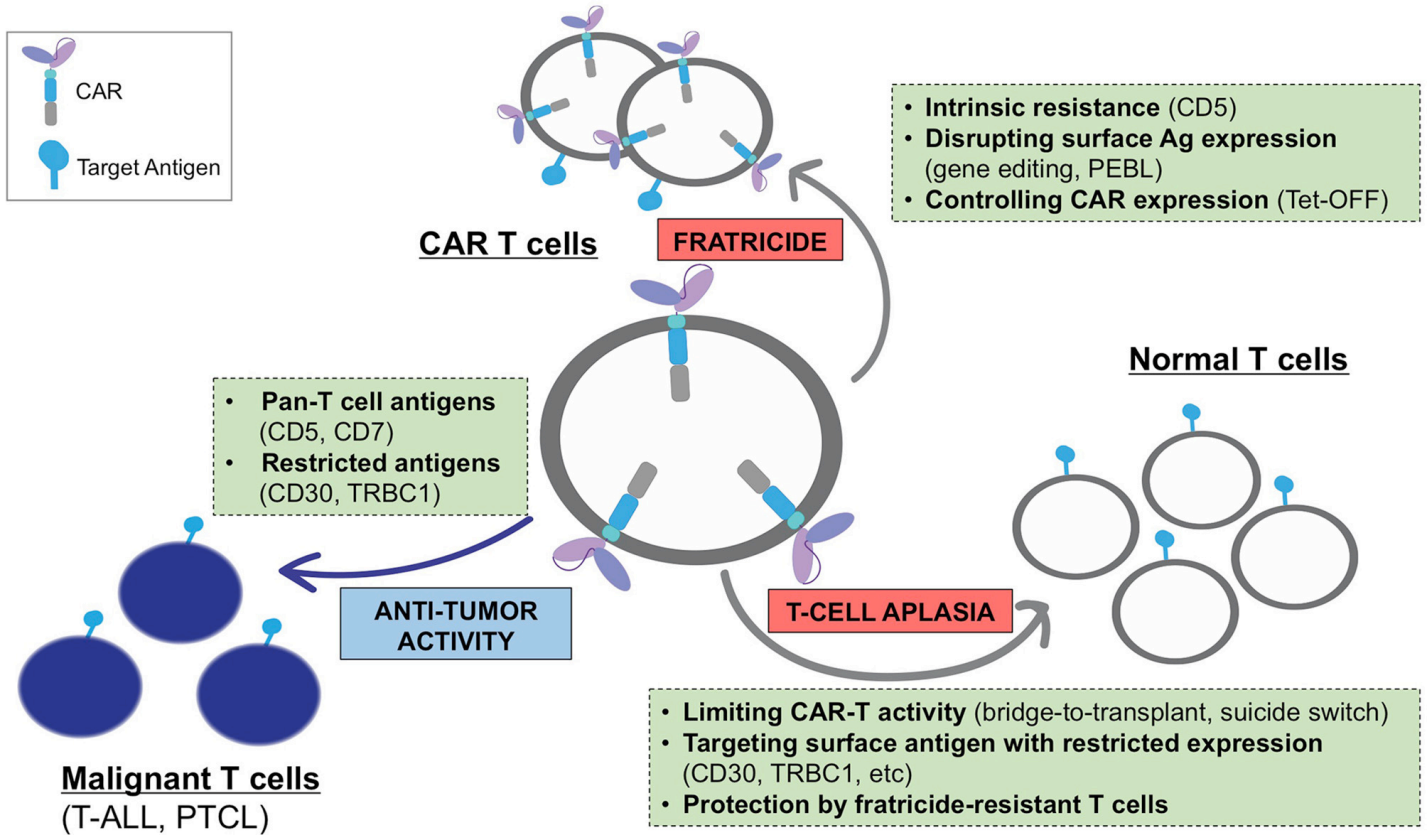
Challenges associated with the development of CARs for T-cell malignancies and main strategies to overcome these limitations.

Expression of a CAR specific to antigens expressed on transduced T cells often results in continuous (tonic) ligand-driven CAR stimulation, potentially causing fratricide of the transduced T cells and accelerating their differentiation to terminal effector subsets with limited *in vivo* persistence. The magnitude of the tonic CAR signaling and the associated toxicities depends on the expression and availability of the target antigen on T cells and can be minimized by: (a) reducing the expression of the target antigens on T cells, (b) regulating the expression and/or activity of the CAR, and (c) using cell subsets naturally lacking expression of the target antigen as a platform for CAR expression (Figure 1).

Targeting a T-cell antigen with more restricted expression (CD30, TRBC1, etc.) limits fratricide only to a subset of antigen-positive T cells and promotes the expansion of the remaining cells with retained CAR-specific cytotoxic function. However, in some cases this mechanism eliminates a T-cell subset with a necessary function (e.g., CD4^+^ helper T cells in CD4 CAR T cells), which may reduce the anti-tumor activity of these CAR T cells. The expansion of fratricide-resistant cells can occur even when targeting a pan-T cell antigen with broad expression. As discussed above, T cells expressing a CD5 CAR became resistant to self-targeting shortly after transduction, likely due to the rapid loss of detectable surface CD5 (39). Notably, fratricide was dramatically increased when T cells expressed a CD5 CAR containing 4-1BB and other TNFR superfamily costimulatory endodomains (97). Continuous CAR signaling promoted the upregulation of the adhesion molecule ICAM-1 and stabilized fratricidal immune synapses between the CD5 CAR T cells, in a 4-1BB-TRAF2 signaling-dependent manner. Therefore, the choice of CAR signaling domains may be important to minimize self-targeting and maximize the therapeutic effect of CARs directed to T-cell antigens.

In contrast to CD5, CARs specific to other pan-T cell antigens such as CD7 and CD3 produce overwhelming fratricide driven by high residual expression of the target antigens, precluding CAR T cell expansion (56–58, 60). Two strategies have been proposed to minimize this fratricide: disrupting the gene encoding the target antigen (CD7 or CD3) using genome editing tools (56, 57, 60), or preventing cell membrane localization of the target antigen (CD7) with an engineered CD7-binding protein anchored in the endoplasmic reticulum (58). Both approaches effectively reduced surface expression of CAR target antigens and minimized T-cell fratricide. Importantly, deletion of CD7 did not affect the short-term effector function of CAR T cells and enabled the expansion of functional CAR T cells with high anti-tumor activity.

Regulating CAR expression can also effectively reduce CAR-T fratricide during *ex vivo* expansion. We have developed a single-vector Tet-OFF gammaretroviral expression system in which the addition of doxycycline after viral transduction minimized the expression of the highly fratricidal 4-1BB-containing CD5 CAR (97). Low CAR expression prevented fratricide and normalized the expansion of transduced T cells, and full CAR expression was restored *in vivo* in the absence of doxycycline, enabling robust anti-leukemic activity. This approach can be used to produce highly fratricidal CAR T cells without the need to disrupt the expression of the target antigen. Further, the *in vivo* persistence of these CAR T cells would be self-limiting due to the re-emergence of fratricidal activity. It would also be of interest to evaluate whether fratricidal CAR activity in T cells could be prevented using pharmacologic agents that reversibly inhibit TCR signaling kinases, such as Lck and ZAP-70. In this setting, CAR T cells would temporarily have reduced cytotoxic activity during their *ex vivo* expansion and quickly regain effector functions after administration to patients.

Finally, fratricide can be avoided altogether by using cytotoxic cells that naturally lack the target antigen expression. For example, an NK cell line expressing a CD3- or CD5-directed CAR can expand and exert specific cytotoxic activity against malignant T cells without the risk of self-elimination (60, 98, 99). However, the anti-tumor potency of NK-92 cells in mouse models of T-cell leukemia/lymphoma was limited and may be further reduced by the lethal irradiation required for the clinical application of these cells. Evidence is emerging that expressing T-cell specific CARs on normal NK cells could serve as a potent alternative (100, 101).

### Strategies to Limit T-Cell Aplasia

A primary limitation of developing CAR T cells for T cell malignancies is ablation of normal T cells by on-target, off-tumor effects, which could cause profound and prolonged T cell aplasia and put the patient at risk for life-threatening infections. This obstacle may limit the use of CAR T cells for T cell malignancies unless treatment is followed by hematopoietic stem cell transplant to allow for immune reconstitution.

One obvious way to avoid T-cell aplasia is to select an antigen with restricted expression on normal lymphocytes, thereby sparing a population of T cells that could confer immunity. This approach, however, is limited to patients with malignancies that express these select target antigens with high frequency. There are currently several approaches to overcoming the aplasia caused by targeting the pan-T cell antigens present on the majority of T-cell malignancies. For example, incorporating a suicide switch into therapeutic T cells has been shown to produce their rapid elimination in the event of toxicities (102); however, this strategy has yet to be evaluated clinically in T cell-targeting CARs. Alternatively, it is possible to select a CAR, like the CD5 CAR, to which normal T cells are resistant to killing. In the case of CD5 CAR T cells, this resistance likely occurs due to the higher inherent resistance of normal T cells to their own cytotoxic mechanisms (39). Finally, our studies show CAR-modified T cells themselves retain TCR function and thus exert a degree of immune protection (56). This finding provides a rationale for developing engineered, fratricide-resistant T-cells, such as gene-edited virus-specific T cells, to use as a platform for CARs or to infuse as a supplementary cell product alongside the CAR T cells. These polyclonal T cells would be gene-modified to resist CAR-directed fratricide and enable systemic protection for the duration of cell therapy, thereby enabling the broader application of CAR T cells targeting T cell malignancies (Figure 1).

### Reducing the Risk of Transducing Malignant Blasts

One risk of adoptive cell therapy using autologous products is the inadvertent incorporation of circulating malignant blasts into the transduced T cell population. Transducing malignant T cells with CARs, and in some cases allowing them to undergo gene editing to knock down expression of the target molecule is a real risk and one that must be factored into any informed consent for administration of these agents. However, given the poor survival and expansion of malignant blasts during the manufacturing process, these unwanted modifications of malignant cells should occur at a low frequency (103). Moreover, in the absence of gene editing to decrease target antigen expression, malignant blasts expressing the target antigen may undergo fratricidal elimination by CAR T cells after transduction and prior to infusion into the patient. Additionally, only some relapsed patients have circulating blasts, further limiting the scope of this problem. For instance, there are minimal to no circulating blasts in most PTCL patients at relapse, and only florid relapse in T-ALL demonstrate circulating blasts, often lacking surface CD3 expression. Therefore, CD3 selection could be implemented to enrich the starting autologous sample for normal T cells and minimize the risk of genetic modification of malignant blasts.

One can entirely avoid the risk of transducing malignant cells by using off-the-shelf allogeneic T cells or NK-cells as vehicles for CAR delivery. Since these cell products would be generated from healthy donors they would also obviate the possibility of using patient-derived T cells that are hypofunctional as a result of exposure to inhibitory tumor microenvironment or to prior intensive therapies. Abrogating surface expression of TCR by disrupting the expression of a TCR subunit is one commonly used strategy to produce a universal CAR-T product (104–106). Indeed, expressing a CD7 CAR on TCR-edited T cells minimized xenogeneic graft-vs.-host disease (GvHD) in mouse models while preserving anti-leukemic activity via the CAR (57). This approach can be used to manufacture banked CD7 CAR T cells from healthy donors and administer them to HLA-unmatched recipients with a minimal risk of GvHD and low potential for immune rejection due to the anticipated toxicity against host CD7^+^ T- and NK-cells. One limitation of this strategy is the potential loss of systemic TCR function due to elimination of endogenous T cells and substitution with TCR-negative CAR T cells, which would compromise viral immunity. An alternative approach is to use CAR-modified multivirus-specific T (multiVST) cells engineered to be fratricide-resistant. These cells can be manufactured from healthy donors and would simultaneously produce anti-tumor activity and, in the case of T-cell aplasia, exert protection against viral infections that commonly arise in T-cell deficient patients (107). Due to the restricted TCR specificity, CAR-expressing multiVST cells demonstrated very low capacity for GvHD and were shown to be safe and effective in immunocompromised patients (108). Therefore, multiVST are a promising platform for the development of off-the-shelf CAR-based therapies for T-cell malignancies.

## Summary and Conclusions

CAR T cell therapies for T-cell malignancies face multiple unique challenges, such as immediate fratricide of CAR T cells *in vitro* and prolonged suppression of endogenous T-cell function *in vivo*. To date, many strategies have been developed to minimize these unwanted events and maximize the antitumor activity of CAR T cells targeted toward T-cell malignancies. Because T-lineage malignancies share many similarities with B-cell tumors, some outcomes and toxicities of T cell-specific CAR T cells may resemble those commonly observed in patients with B-cell malignancies e.g., on-target off-tumor toxicities of CAR T cells, emergence of antigen escape tumor clones. This phenomenon of antigen escape may arise even when targeting widely expressed T-cell antigens given the considerable heterogeneity between tumor cell lines in T-cell malignancies. Combinatorial antigen targeting may limit the selection of antigen-negative clones and thus can be a future consideration in T-cell immunotherapy if such relapses are seen. Currently, several ongoing and upcoming first-in-man clinical studies will evaluate the safety and efficacy of autologous and allogeneic CAR T cells in patients with T-cell leukemia and lymphoma, both as a “bridge-to-transplant” and a standalone therapy outside the transplant setting. Ultimately, these results will inform the design of next-generation therapies, which may well also include additional T cell engineering strategies to increase resistance to tumor immune evasion strategies. Ultimately, the field will likely progress toward allogeneic banked therapeutic cells, engineered for tumor resistance and combinatorial antigen targeting.

## Author Contributions

LS and MM wrote and edited the manuscript. MB contributed to manuscript planning and editing.

### Conflict of Interest Statement

The authors declare that the research was conducted in the absence of any commercial or financial relationships that could be construed as a potential conflict of interest.
